# Automated FiO_2_-SpO_2_ control system in Neonates requiring respiratory support: a comparison of a standard to a narrow SpO_2_ control range

**DOI:** 10.1186/1471-2431-14-130

**Published:** 2014-05-28

**Authors:** Maria Wilinska, Thomas Bachman, Janusz Swietlinski, Maria Kostro, Marta Twardoch-Drozd

**Affiliations:** 1 Neonatology, The Medical Centre of Postgraduate Education, Marymoncka 99/103, Warsaw 01-813, Poland; 2 Economedtrx, PO Box 1269, Lake Arrowhead, CA 92352, USA; 3 Ruda Slaska City Hospital, Ruda Slaska, Poland

## Abstract

**Background:**

Managing the oxygen saturation of preterm infants to a target range has been the standard of care for a decade. Changes in target ranges have been shown to significantly impact mortality and morbidity. Selecting and implementing the optimal target range are complicated not only by issues of training, but also the realities of staffing levels and demands. The potential for automatic control is becoming a reality. Results from the evaluation of different systems have been promising and our own experience encouraging.

**Methods:**

This study was conducted in two tertiary level newborn nurseries, routinely using an automated FiO_2_-SpO_2_ control system (Avea-CLiO2, Yorba Linda CA, USA). The aim of this study was to compare the performance of the system as used routinely (set control range of 87-93% SpO_2_), to a narrower higher range (90-93%). We employed a 12-hour cross-over design with the order of control ranges randomly assigned for each of up to three days. The primary prospectively identified end points were time in the 87-93% SpO_2_ target range, time at SpO_2_ extremes and the distribution of the SpO_2_ exposure.

**Results:**

Twenty-one infants completed the study. The infants were born with a median EGA of 27 weeks and studied at a median age of 17 days and weight of 1.08 kg. Their median FiO_2_ was 0.32; 8 were intubated, and the rest noninvasively supported (7 positive pressure ventilation and 6 CPAP). The control in both arms was excellent, and required less than 2 manual FiO2 adjustments per day. There were no differences in the three primary endpoints. The narrower/higher set control range resulted in tighter control (IQR 3.0 vs. 4.3 p < 0.001), and less time with the SpO_2_ between 80–86 (6.2% vs. 8.4%, p = 0.006).

**Conclusions:**

We found that a shift in the median of the set control range of an automated FiO_2_-SpO_2_ control system had a proportional effect on the median and distribution of SpO2 exposure. We found that a dramatic narrowing of the set control range had a disproportionally smaller impact. Our study points to the potential to optimize SpO_2_ targeting with an automated control system.

## Background

Managing SpO_2_ to a target range rather than just increasing FiO_2_ in response to an episode of desaturation became the standard of care more than a decade ago. Subsequently, shifting of SpO_2_ target ranges was shown to have important impact on outcomes [1-3]. Most importantly, recent large multicenter trials have shown that pulmonary and retinal morbidity can be reduced, by lowering the SpO_2_ target range, but also that lowering it too low, increases mortality [4-7]. It was reported some time ago that lower SpO_2_ target ranges are also associated with an increased rate of desaturations [8]. It has been recently speculated that this effect might explain the increased mortality seen in trials of lower target ranges [9].

Clinically applying the results of this targeting evidence is challenging because of the difference between the SpO_2_ target range specified and the SpO_2_ exposure actually achieved. Furthermore selecting the optimal clinical SpO_2_ target range is additionally complicated by the realities of manual titration of FiO_2_ in the busy newborn ICU [10-12]. That is, the selection of the clinical SpO_2_ target range must take into account what is practical.

Automated closed loop control systems have been proposed, studied and shown to be effective [13-16]. One is commercially available, and has been in routine use in our units for two years. (AVEA-CLiO2, CareFusion, Yorba Linda CA, USA). Such systems can make adjustments to FiO_2_ as often as every second. This is in contrast to nurses responding to persistent alarms and making adjustments, generally within minutes. Automation of SpO_2_-FiO_2_ control, because it does not require constant nursing intervention, also makes it practical to consider different paradigms for management of SpO_2_ exposure.

Our aim was to compare, using a crossover design, the relative effectiveness of our automated SpO_2_-FiO_2_ system set at two difference control ranges. The first was our standard SpO_2_ target range of 87-93% and the second, a narrower range with a higher midpoint (90-93%). The former was selected with the idea that lower SpO_2_ associated with the increased risk of desaturations might be reduced.

## Methods

The study was conducted in two tertiary care neonatal centers in Poland (The Medical Center of Postgraduate Education, Warsaw and City Hospital, Ruda Slaska).

Our research was approved by the The Ethics Committee of the Medical Centre of Postgraduate Education. Written informed consent was obtained from the parents of each patients.

In this study we use the term set control range to describe the settings on the automated SpO_2_-FiO_2_ control system and the term target range to describe the desired clinical target range for SpO_2_. The study was a crossover design, where subjects were switched between the two set control ranges, every 12 hours for 3 days. One range was that used routinely in the unit (87-93% SpO_2_) the other was 90-93% SpO_2_. To avoid the concern that different levels of nursing staffing and frequency of procedures in the evenings might effect the relative oxygenation stability, the change was made midday. The order was randomized for each day. For analysis, each subject’s experience over the study at each set control range was averaged together. Digital SpO_2_ and FiO_2_ data were collected every 5 seconds from the ventilator. Infants were enrolled if the research staff and data collection system were available, if they had exhibited at least 4 desaturations <80% SpO_2_ in the preceding 8 hours, and if they were expected to remain on their current mode of respiratory support (invasive or noninvasive) for the 3-day study period.

The prospectively selected primary endpoints were % time in our standard 87-93% SpO_2_ target range, % time at SpO_2_ extremes (SpO_2_ < 80% or >98%) and the % time with SpO_2_ between 80-86%. Time when SpO_2_ was over the SpO_2_ target range, but the FiO_2_ was 0.21, was included in the time in SpO_2_ target range. We hypothesized that the narrower SpO_2_ range would result in more time in the 87-93% SpO_2_ range, comparable time at SpO_2_ extremes and less time with SpO_2_ between 80%-86%. Based on data from a pilot study, we projected a sample size of 20 subjects would be able to detect a 3% difference in the time in the target range and low SpO_2_ range (power >90%, p < 0.05) and also a 1% difference in the SpO_2_ extremes (power >80%, p < 0.05).

All the primary and descriptive endpoints were continuous variables. Evaluation of the paired differences between the two set control ranges were tested with the Andersen-Darling test for normality. When a normal distribution was not present, a Wilcoxon Signed Rank Test was used to evaluate paired differences. Otherwise two tailed paired t-tests were used. A p < 0.05 was considered statistically significant for all comparisons. A post hoc evaluation of the relationship between % time in the target range and rate of severe desaturations was explored with linear regression and correlation. All the statistical tests were conducted with SigmaXL version 6.1 (Toronto, Canada).

## Results

The study was conducted between November 2011 and February 2013. Twenty-four infants were enrolled. Three experienced clinical changes unrelated to the study and did not complete more than 1 day of study. They were excluded. Eighteen completed 3 days and 3 completed 2 days. Of these 21 infants, 8 were intubated, 6 received nasal positive pressure ventilation and 7 nasal continuous airway pressure. The baseline characteristics [median(IQR)] of the subjects were, EGA: 27 weeks (26–29), study weight: 1.08 kg (0.86-3.65), age: 17 days (1–26) and FiO_2_: 0.32 (0.24-0.90).

**Figure 1:**
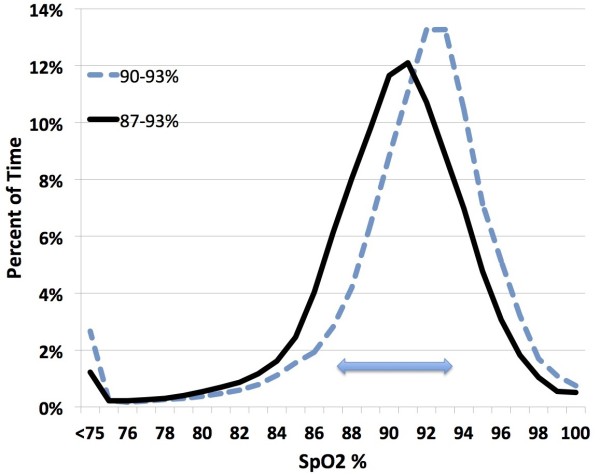
**Histogram of SpO2 exposure for the two Control Ranges.** The bar highlights the standard target range (87-93% SpO_2_).

During the study period a significant desaturation (SpO_2_ < 80%) occurred about every 30 minutes. Among the subjects, the incidence of these significant desaturations ranged between every 120 minutes and every 10 minutes. The need to manually adjust FiO2 was uncommon (<2 per day). The histogram of the SpO_2_ exposure for the two control settings is seen in Figure 1. There is a clear difference. The mean of the SpO2 medians during 90-93% control, which also had a higher midpoint, was correspondingly higher than the median for the standard 87-93% SpO_2_ control range (91.9% vs 90.7% p < 0.001). The interval of the IQR of SpO_2_ associated with the 90-93% control range was narrower (3.0% vs 4.3% p < 0.001).

The primary endpoints are tabulated in the Table 1. Times in the 87-93% SpO_2_ target range, and at SpO_2_ extremes were clinically comparable, and not statistically significantly different. However there was a trend suggesting less time with SpO_2_ < 80% during 87-93% control. The median time with SpO_2_ between 80-86% was statistically significantly less (6.2% vs 8.4%, p = 0.006) during periods when the control range was set at 90%-93%. However, as is suggested in Figure 1, the higher narrower set range resulted in more time with SpO_2_ between 94-98% (16.4% vs 12.0%, p = 0.005).

**Table 1:** The primary endpoints

	**87-93**	**90-93**	**p**
Time* in 93-87% SpO_2_	72.9% (16.8)	71.3% (16.4)	0.813
Time <80% SpO_2_	1.6% (3.4-0.7)	1.7% (3.1-0.5)	0.052
Time* >98% SpO_2_	0.4% (0.7-0.1)	0.4% (1.9-0.2)	0.102
Time 86-80% SpO_2_	8.4% (11.4-6.8)	6.2% (10.3-2.8)	0.006

Data mean(stdev) or median(IQR). p from paired Wilcoxon Sign Rank test for medians and paired t-test for means, *% Time >93% when FiO_2_ = 0.21 is treated as in 87-93%.

There were no clinically relevant differences between the two set control ranges (90-93%, 87-93%) in the median frequency of desaturations <80% SpO_2_ (1.7/hour vs. 1.9/hour, p = 0.25), or the median duration of those episodes <80% SpO_2_ (16.4 seconds vs. 14.8 seconds, p = 0.57).

The percent time with SpO_2_ between 87-93% for these subjects varied widely (34% to 94%). Time in this target range was highly correlated (R squared = 0.720) to the frequency of desaturations <80% and decreased 2.4% with every added desaturation/hour. This relationship was nearly identical for the two control ranges.

## Discussion

We compared the relative effectiveness of automated control of SpO_2_-FiO_2_ set at two control ranges. One range reflected our clinical practice at the time (87-93% SpO2) for both manual and the automated SpO_2_-FiO_2_ system control. The second was narrower with a higher midpoint (90-93% SpO_2_). We found that both set control ranges were effective, but that there were differences. The narrower range did not increase time in the intended target range (87–93 SpO_2_) or markedly impact time at SpO_2_ extremes (<80%, >98%). The narrow range, with a higher midpoint, did result in less time with lower SpO_2_ (80-86%), more time with higher SpO_2_ (94-98%) and also a tighter distribution of the SpO_2_.

This is, we believe, the first report of a comparison of the relative effectiveness of two automated control ranges. We suggest that our study demonstrates the potential to use automated control ranges that are narrower or shifted to impact changes in SpO_2_ exposure. In this study we were able to reduce the time below the desired SpO_2_ target range, provide tighter control, but not increase time in the SpO_2_ target range. The latter was a result of more time above the target range. We have since participated in a large multicenter study that was completed in early 2014 [17]. This multicenter study is not only comparing two narrow target ranges, but also the relative effectiveness of automated SpO_2_-FiO_2_ system and manual control for the two ranges. We hope that the results of this new study, when considered with our findings, will result in better insight into optimum automated targeting strategies.

Claure validated the relative effectiveness of CLiO2 as compared to manual control in two studies [13,14]. In both these studies automated SpO_2_-FiO_2_ system control resulted in more time in the designated SpO_2_ target range and less time with SpO_2_ > 98%, as compared to manual control. However, both of these studies also showed that automated SpO_2_-FiO_2_ control was associated with more time below the target range. We speculated that this effect was a result of a shift in distribution related to the higher median SpO_2_ experienced during manual control. In our study a shift in the median SpO_2_ between the two set control ranges also resulted in a reduction in the time with SpO_2_ between 80-86%. In our study the range with a higher median SpO_2_ also was narrower and resulted in a tighter distribution. The latter would also have reduced the time between 80%-86% SpO_2_. It is not possible, therefore, to determine from our data to what degree these two factors were causal. However we speculate that a narrower control range with the same mid point as the desired target range would result in optimum results.

McEvoy demonstrated in 21 intubated infants with chronic lung disease that a lower level of baseline SpO_2_ control resulted in a tripling of the incidence of significant desaurations [8]. In evaluating the evidence of increased mortality associated with lower SpO_2_ target range, it has been speculated that this effect might be the cause of increased mortality in infants managed with a low target range [9]. In our study we found no difference between the rate of severe desaturations associated between the two assigned ranges. In our study, while both control ranges had similar time in the desired SpO_2_ target range, the 90-93% range resulted in a higher median SpO_2_, and less time below the SpO_2_ target range and 80% SpO_2_. In McEvoy’s study there was a much more marked difference in both the median SpO_2_ and time below the SpO_2_ target range. We feel this bigger difference likely explains the difference between our results. We further suggest that the ability of automated SpO_2_-FiO_2_ control to reduce the time below the SpO_2_ target range, might reduce the risk of this problem as seen in McEvoy’s study and other studies of SpO_2_ targeting.

In a previous crossover study we compared CLiO2 with two difference manual control strategies in 15 infants during 8-hour test periods [18]. We found that the relative effectiveness of automated control was much more pronounced in infants experiencing frequent severe desaturations. For this reason, on a post hoc basis, we explored the effect of desaturation rate and time in the SpO_2_ target range. We found a linear relationship of decreasing time in the SpO_2_ target range to increasing rate of severe desaturations, which is consistence to the effect seen in our previous study.

While offering great promise for increased effectiveness, automation of FiO_2_ control also dramatically reduces the need for nursing intervention, with significant potential for labor savings. We reported that manual adjustments of FiO_2_ occurred less than twice per day. In a multicenter trial, Claure reported 10 FiO_2_ adjustments per day during automated control, a decrease of over 90% compared to manual control [14]. In contrast Hallenberger, from the multicenter comparison of a different FiO_2_-SpO_2_ control system, reported that FiO_2_ adjustments were made 52 times per day during automated control, a decrease of 32% compared to manual control [15]. Both studies reported a wide variation among patients, as was also the case in our study. The system studied by Hallenberger has implemented a different approach to FiO_2_ control, that would by its nature require more nursing intervention. The difference between our experience of less than 2 per 24 hours and that reported by Claure is primarily a result of the stability of the infants being studied, we believe. That is, there was a wide difference in the FiO_2_ level, the rate of severe desaturations and time in the target range between the study populations. This is only a speculation, as factors such as the SpO_2_ alarm settings and general attentiveness of the staff might also be related.

Our study has some limitations. It was a relatively small study of 21 infants. Still it represents almost 1500 hours of automated control, nearly twice that reported in the largest prior study of CLiO2 [14]. In addition we saw a trend toward more time <80% SpO_2_ in one intervention, a larger sample with more power would have clarified the relevance of this trend. The study population was also diverse in terms of frequency of severe desaturations, weight and mode of respiratory support. We feel that this is an advantage as it reflects the typical intended population for use. However the study was not powered to explore relative differences in among these categories. Finally we studied an SpO_2_ range that was both narrower and higher, confounding the interpretation of the effect of these two parameters.

## Conclusions

We found that a shift in the median of the set control range of CLiO2 had a proportional effect on the median and distribution of SpO_2_ exposure. We found that a dramatic narrowing of the set control range, had a disproportionally smaller impact. Our study points to the potential to optimize SpO_2_ targeting with an automated FiO2-SpO2 control system.

## Abbreviations

CPAP: Continuous positive airway pressure; BPD: Bronchopulmonary dysplasia; EGA: Estimated gestational age; FiO2: Fraction of inspired oxygen; ROP: Retinopathy of prematurity; SpO2: Percent saturation of oxygen as measured by pulse oximetry.

## Competing interests

TB is an independent clinical research consultant and provides services to the manufacturer of the system tested in this study. However, this project was not funded in anyway by the manufacturer, nor were they involved in it conception or design or oversight. The other authors declare they have no competing interests.

## Authors’ contributions

All authors (MW, TB, JS, MK, MT-D) critically reviewed, edited and approved the submitted manuscript. TB conducted the analyses and drafted the manuscript. TB also proposed the need for the study. MW, TB and JS were involved in the development of the aims and methods of the study. MW, directed all aspects of the study. MK and MT-D were responsible for screening the subjects, oversight of the clinical care during the study and collection of all study data.

## Authors’ information

TB is a retired healthcare administrator and university instructor. He continues to collaborate on a pro bono basis with former colleagues on a variety of respiratory research projects. In addition he provides, through his consultancy Economedtrx, clinical research services. MW, JS, MK and MT-D are involved neonatal care. MW and JS are also actively engaged in education and research.

## Pre-publication history

The pre-publication history for this paper can be accessed here:

http://www.biomedcentral.com/1471-2431/14/130/prepub
